# The Link between NAFLD and Metabolic Syndrome

**DOI:** 10.3390/diagnostics13040614

**Published:** 2023-02-07

**Authors:** Fabiana Radu, Claudia-Gabriela Potcovaru, Teodor Salmen, Petruța Violeta Filip, Corina Pop, Carmen Fierbințeanu-Braticievici

**Affiliations:** 1 Doctoral School, Carol Davila University of Medicine and Pharmacy, 050474 Bucharest, Romania; 2 Department of Gastroenterology and Internal Medicine, Clinical Emergency University Hospital, 050098 Bucharest, Romania; 3 Department of Gastroenterology, Emergency University Hospital Bucharest, 050098 Bucharest, Romania

**Keywords:** metabolic syndrome, insulin resistance, NAFLD, early diagnosis, hepatocellular carcinoma

## Abstract

Metabolic syndrome (MetS) is characterized by an association of cardiovascular and diabetes mellitus type 2 risk factors. Although the definition of MetS slightly differs depending on the society that described it, its central diagnostic criteria include impaired fasting glucose, low HDL-cholesterol, elevated triglycerides levels and high blood pressure. Insulin resistance (IR) is believed to be the main cause of MetS and is connected to the level of visceral or intra-abdominal adipose tissue, which could be assessed either by calculating body mass index or by measuring waist circumference. Most recent studies revealed that IR may also be present in non-obese patients, and considered visceral adiposity to be the main effector of MetS’ pathology. Visceral adiposity is strongly linked with hepatic fatty infiltration also known as non-alcoholic fatty liver disease (NAFLD), therefore, the level of fatty acids in the hepatic parenchyma is indirectly linked with MetS, being both a cause and a consequence of this syndrome. Taking into consideration the present pandemic of obesity and its tendency to drift towards a progressively earlier onset due to the Western lifestyle, it leads to an increased NAFLD incidence. Novel therapeutic resources are lifestyle intervention with physical activity, Mediterranean diet, or therapeutic surgical respective metabolic and bariatric surgery or drugs such as SGLT-2i, GLP-1 Ra or vitamin E. NAFLD early diagnosis is important due to its easily available diagnostic tools such as non-invasive tools: clinical and laboratory variables (serum biomarkers): AST to platelet ratio index, fibrosis-4, NAFLD Fibrosis Score, BARD Score, fibro test, enhanced liver fibrosis; imaging-based biomarkers: Controlled attenuation parameter, magnetic resonance imaging proton-density fat fraction, transient elastography (TE) or vibration controlled TE, acoustic radiation force impulse imaging, shear wave elastography, magnetic resonance elastography; and the possibility to prevent its complications, respectively, fibrosis, hepato-cellular carcinoma or liver cirrhosis which can develop into end-stage liver disease.

## 1. Introduction

Metabolic syndrome (MetS), also known as insulin resistance (IR) syndrome or Syndrome X (terminology not commonly used as another syndrome X has been documented in cardiology), represents an association of risk factors for cardiovascular (CV) disease (CVD) and type 2 diabetes mellitus (T2DM) that co-occur more frequently than by chance. These risk factors are represented by high blood pressure (HBP), impaired fasting glucose (IFG), increased level of triglycerides (TG), low high-density lipoprotein (HDL) cholesterol levels, and obesity (mostly abdominal type). It is becoming increasingly clear that this constellation of metabolic disorders is connected to IR and is more frequently found in people with abdominal obesity, particularly in those with an excess of intra-abdominal or visceral adipose tissue [1]. IR creates an atherogenic, inflammatory and prothrombotic state and it is not only a factor which increases the risk of T2DM but also a prevalent cause of CVD [2]. Non-alcoholic fatty liver disease (NAFLD) is strongly related with IR, and it can be a cause, but also a consequence of MetS [3]. It is estimated that 32.4% of the population worldwide has NAFLD. The incidence and prevalence have rapidly increased over time, from 25.5% before 2005 to 37.8% in 2016 [4], synchronising with the global obesity pandemic [5] and becoming one of the leading causes of cirrhosis in some countries [6]. Moreover, it is predicted that, in terms of indication for liver transplantation, NAFLD will exceed the viral ethology [7]. The overall prevalence of NAFLD was significantly higher in male than in female. Liver biopsy is the gold standard for diagnosis NAFLD, but due to its inconvenience, other non-invasive ways of diagnosis were developed (serum biomarkers and imaging-based biomarkers).

This article aims to assess the relationship between the MetS and development of NAFLD, as well as the diagnostic procedures for NAFLD, in order to ensure that this condition is identified and treated promptly as to avoid complications such as fibrosis or hepatocellular carcinoma (HCC), which are an important economic burden, but also, in the long run, lead to the patient’s disability, and, therefore, higher costs for the healthcare systems are required.

## 2. Definition of MetS

The challenge represented by MetS is that various organizations offered slightly different clinical screening parameters and cut-off values for identifying individuals with MetS which are somewhat ambiguous compared to the conceptual description of the MetS. The first formalized definition of MetS was offered in 1998 from a consultation group towards the World Health Organisation (WHO). The WHO diagnosis has mandatory markers of IR (glucose ˃ 6.1 mmol/L or > 110 mg/dL, 2 h glucose ˃ 7.8 mmol/L or > 140 mg/dL) and a minimum of two additional risk factors: low HDL-cholesterol level (HDL-cholesterol ˂ 0.9 mmol/L or < 35 mg/dL in males and ˂ 1.0 mmol/L or < 40 mg/dL in females), high TG levels (˃1.7 mmol/L or >150 mg/dL), obesity (waist/hip ratio ˃ 0.9 in male or ˃ 0.85 in female or body mass index (BMI) ˃ 30 kg/m^2^), HBP with systolic (˃140/90 mmHg). In 2001, the National Cholesterol Education Program Adult Treatment Panel III (ATP) [8] developed a new definition for MetS that did not require the expression of IR or a single factor for diagnosis, but rather the presence of 3 out of the 5 factors listed below which include abdominal obesity (waist ˃ 102 cm in males or ˃ 88 cm in females) (which is strongly associated with IR), elevated TG (˃1.7 mmol/L or >150 mg/dL), reduced HDL cholesterol (˂1.0 mmol/L or <40 mg/dL in males, ˂1.3 mmol/L or <50 mg/dL in females or drug treatment for low HDL cholesterol), elevated blood pressure (BP) (˃130/85 mmHg or drug treatment for HBP), and IFG (glucose > 5.6 mmol/L or > 100 mg/dL or drug treatment for elevated blood glucose) (IFG or T2DM). To integrate these two different definitions, the International Diabetes Federation (IDF) [9] and the American Heart Association/National Heart, Lung, and Blood Institute (AHA/NHLBI) [10] promulgated in 2005 a new definition for MetS, but there were variations based on waist size. IDF, in comparison with WHO’s definition, assert abdominal obesity as mandatory (waist ˃ 94 cm in males or ˃ 80 cm in females) along with the presence of two or more of the following: blood glucose > 5.6 mmol/L (100 mg/dL) or diagnosed DM, HDL cholesterol ˂ 1.0 mmol/L (<40 mg/dL) in males, ˂1.3 mmol/L (<50 mg/dL) in females or drug treatment for low HDL cholesterol, blood TG ˂ 1.7 mmol/L (<150 mg/dL) or drug treatment for elevated TG, BP ˃ 135/85 mmHg or drug treatment for HBP. AHA/NHLBI did not consider abdominal obesity as mandatory, and the waist parameters are 102 instead of 94 in males and 88 instead of 80 in females. The parameters of the waist circumference (WC) from AHA/NHLBI are indicators for a BMI of approximately 30 kg/m^2^, and those from IDF are more suggestive of a BMI of 25 kg/m^2^. Although they are not as widely used, somewhat slightly different definitions were utilized by other organizations such the European Group for the Study of IR (EGIR) and the American Association of Clinical Endocrinologists (AACE) in 2003. Because of its heterogenous definition and cut-off criteria, the data existing on the epidemiology of MetS differs. It is observed that the prevalence of MetS is higher using AHA and IDF (more sensitive criteria) compared to the ATP III definition, with a ranging prevalence from 12.5% to 31.4% worldwide [11].

## 3. MetS and Liver Involvement

Initially, Reaven did not include obesity in his description of syndrome X since he could identify non-obese people with IR and people with obesity who were insulin sensitive. Obesity assessed by BMI alone, is not a predictor of MetS if not correlated with WC, age, gender, and ethnicity, because IR has a strong connection with visceral adipose tissue. It is important to identify people with adipose tissue that is distributed in deep compartments because adipocytes from deep compartments are more metabolically active compared with superficial adipocytes and are correlated with IR [12]. In this context, studies evaluating adiposity using computed tomography (CT) revealed that an excessive build-up of visceral adipose tissue was a key correlate of the characteristics of IR; however, CT is unlikely to be employed widely due to the radiation exposure and cost of use [13]. More recently, anomalies that are clustered together, as with those seen in visceral obesity, were found to be nearly identical in people with extra liver fat. Liver fat accumulation may be evaluated non-invasively and with high precision thanks to the development of magnetic resonance spectroscopy. The data provided by this method corresponds with the findings of liver biopsies, making it the best non-invasive way to determine the hepatic triglyceride content (HTGC) and to identify hepatic steatosis [14]. Liver fat is tightly associated with fasting insulin concentrations and direct measurements of hepatic insulin sensitivity, while the amount of subcutaneous adipose tissue is not [15]. A fatty liver overproduces glucose and lipids, especially very low-density lipoproteins (VLDL), the main players of MetS, but also the majority of the well-known CV risk factors, including fibrinogen, C-reactive protein (CRP), plasminogen activator inhibitor-1 (PAI-1) and coagulation factors [15,16].

## 4. NAFLD in the Pathogenesis of Metabolic Syndrome

NAFLD refers to the presence of fat in the liver (˃5–10% of hepatocytes are fatty) which is not associated with other known causes of steatosis such as: alcohol (defined as >20 g of alcohol daily for females and >30 g for males in European and American recommendations [17]), viruses, drugs, toxins, autoimmune disease or iron overload and is accompanied most frequently, if not always, by IR [18]. NAFLD ranges from simple fatty infiltration, without evidence of inflammation (non-alcoholic fatty liver (NAFL)), to fat and inflammation (non-alcoholic steatohepatitis (NASH)) and cirrhosis, which can progress to end-stage liver disease (ESLD) or directly to HCC. It is not a rule that all subjects with MetS develop NAFLD, nor do all subjects with NAFLD develop NASH [19]. NAFLD and MetS may be linked in a vicious cycle, with NAFLD becoming both a symptom and a cause of MetS [20]. From the histological point of view, alcoholic steatohepatitis is indistinguishable from NASH and is characterized by small and large macrovesicular steatosis droplets, but may also be composed of a mixture of large and small vacuoles, ballooning necrosis, mild inflammation, fibrosis, and it can be diagnosed by identifying these features on biopsy liver samples as a gold standard method [21]. In the USA, NASH is the third most common reason for liver transplantation [22] and the most common cause of cryptogenic cirrhosis. According to reviews, 3–6% of people globally are thought to have NASH. It can proceed to cirrhosis and ESLD, even though CVD is the primary cause of mortality in persons with this condition [23]. In addition, the percentage of individuals in the United States who have NASH as the primary cause of their HCC has increased 7.7-fold (from 2.1 to 16.2%) [24]. NAFL is typically asymptomatic, and most patients have normal transaminases levels, even though the disorder is the most common reason for unexpectedly elevated transaminase. At this time, it is not recommended to screen asymptomatic people or people with MetS or simple steatosis [18]. According to a recent assessment, NAFLD may be responsible for 80% of cases of increased transaminase levels in the American population [25] and similar data have been obtained in the Italian [26] and Japanese [27] population. Due to the increase in Western industrialized food, and the sedentary lifestyle, there is an obesity epidemic beginning in childhood with the increase of DM and, respectively, an increase in NAFLD which is now recognized as the most prevalent chronic liver disease, with a worldwide prevalence of 25% [28], with a high prevalence rate reaching 60–70% in patients with DM [29]. T2DM is the most important risk factor for NAFLD and seems to be correlated with the progression of the disease, with the presence of advanced fibrosis (AF), and is associated with liver-related mortality, but it is usually overlooked in clinical practice [30].

## 5. New Therapeutic Perspectives in the Association between NAFLD and MetS

Montemayor et al. reported [31] in a study on 128 patients aged 40–60 years with BMI between 27 and 40 kg/m^2^, with diagnosis of NAFLD and MetS, that conventional diet (CD) 43 patients, Mediterranean Diet (MD)—high meal frequency, 43 patients, and MD—physical activity (PA), 42 patients, decreased the intrahepatic fat content and liver stiffness alongside with BMI, insulin, HbA1c, diastolic BP, HDL-C and ALT after 12 months as seen in Table 1.

Konieczna et al. [32] reported in a study on 5867 patients with NAFLD risk factors who used to consume ultra-processed foods, that administration of energy-restricted MD, PA and behavioural support decreases the BMI, WC, HbA1c, TG after one year. The dietary intake and PA were assessed at 0, 6 and 12 months using a semi-quantitative food frequency questionnaire and validated Minnesota-REGICOR short PA questionnaire as seen in Table 1.

Van Kleef et al. [33] reported in a study of 667 patients, 229 of which had NAFLD (assessed by ultrasound (US) after excluding secondary causes of liver steatosis such as excessive alcohol consumption, steatogenic drug use, and hepatitis B or C), a mean age of 63.3 years and were females 53%. Of these, 229 (34.3%) had NAFLD and associated a higher prevalence of overweight, DM, HBP, and lipid abnormalities. The intervention consisted in PA—61.9% light intensity, 29.8% moderate intensity, and 8.2% vigorous intensity; and resulted in amelioration of NAFLD incidence and in WC as seen in Table 1.

Recently, Lassailly et al. [34] reported long-term outcomes in a group of 180 patients with severe obesity and biopsy-confirmed NASH who underwent metabolic and bariatric surgery (MBS). At the 5-year post-surgical follow-up, NASH was resolved without deteriorating fibrosis in 84% of patients, fibrosis was reduced compared to baseline in 70.2%, and it was totally resolved in 56%. MBS is now restricted to adults with a BMI of at least 35 kg/m^2^, a clinical indication that excludes many NAFLD patients as seen in Table 1.

In a study of 40 patients with obesity who underwent bariatric surgery, Pedersen et al. [35] concluded that bariatric surgery reduces NAFLD and can reverse NASH. The study divides the patients in two subgroups that were treated with two different surgical methods: 16 patients underwent Roux-en-Y gastric bypass (RYGB), and 24 patients underwent sleeve gastrectomy (SG). RYGB appeared to minimize hepatic steatosis and enhance the plasma lipoprotein profile better than SG. Even though there are presently no NAFLD guidelines evaluating the efficacy of bariatric surgery in treating NAFLD, SG looks to be an equally good alternative to RYGB in bariatric patients with NAFLD as seen in Table 1.

Newsome et al. [36] studied the efficacy of three different dosages of semaglutide once daily (0.1, 0.2, and 0.4 mg) vs. placebo in a large cohort of 320 patients with NASH, aged 18–75 years, 61% female, and 62% with T2DM. NASH resolution was observed in 59% of volunteers treated with the highest dose of semaglutide, compared to 17% in the placebo group. The combined endpoint of NASH resolution and fibrosis improvement was reported in 37% vs. 15% of semaglutide vs. placebo-treated patients as seen in Table 1.

Regarding the other novel antidiabetic non-insulinic drug [37], Mirarchi et al. [38] reported in a review of 13 studies the benefits of sodium-glucose cotransporter-2 inhibitors in patients with NAFLD decrease liver fat content, AST/ALT levels and liver stiffness as seen in Table 1.

**Table 1:** Novel therapeutic approaches in NAFLD and MetS.

Author	Study Groups	Intervention	Outcome
Montemayor et al. [31]	128 patients	Conventional Diet, Mediterranean diet (MD)–high meal frequency MD–physical activity groups.	↓ intrahepatic fat contents ↓ liver stiffness ameliorated BMI, insulin, Hb1Ac, diastolic blood pressure, HDL-cholesterol, and ALT
Konieczna et al. [32]	5867 patients	Energy-restricted MD, physical activity promotion and behavioral support	↓ of BMI, waist circumference ↓ HbA1c ↓ TG
Van Kleef et al. [33]	667 patients	Different intensities of physical activity	↓ NAFLD ↓ waist circumference
Lassailly et al. [34]	180 patients	Bariatric surgery	↓ fibrosis reversed NASH
Pedersen et al. [35]	40 patients	Roux-en-Y gastric bypass sleeve, gastrectomy	↓ NAFLD reversed NASH
Newsome et al. [36]	320 patients	semaglutide	↓ fibrosis resolution of NASH
Mirarchi et al. [38]	Review with 511 patients from 13 studies	SGLT-2i	↓ liver fat content ↓ AST/ALT ↓ liver stiffness.
Vilar-Gomez et al. [39]	180 patients	Vitamin E	↓ overall mortality and transplant rates ↓ rates of hepatic decompensation ↓ risk of death ↓ need for transplant Benefits both in patients with or without T2DM

↓—decrease; BMI—body mass index; HbA1c—glycated haemoglobin; HDL-cholesterol—high density lipoprotein-cholesterol; ALT—alanine aminotransferase; TG—triglycerides; NAFLD—nonalcohol fatty liver disease; NASH- non alcohol steatohepatitis; SGLT-2i—sodium-glucose loop transporter 2; AST—aspartate amino transferase; T2DM—type 2 diabetes mellitus.

About Vitamin E use in liver involvement, Vilar-Gomez et al. [39] reported in a study on 180 with biopsy-proven NASH and bridging fibrosis or cirrhosis—90 that received treatment with vitamin E and 90 matched patients without vitamin E treatment; that vitamin E treatment increase transplant-free survival rates and and lower rates of hepatic decompensation, risk of death or need for liver transplant. The benefits were present both in patients with or without T2DM as seen in Table 1.

## 6. Diagnosis of NAFLD

As we previously emphasized, NAFLD is an entity which includes both NAFL and NASH; the difference between them being the presence of different grades of inflammation and architectural remodelling, for example, fibrosis. Rapid detection of individuals with severe fibrosis is essential for clinical therapy since they have a higher risk of developing life-threatening complications such as HCC or oesophageal varices. To diagnose NAFL, it is important to confirm the presence of hepatic steatosis either by imaging or histology, but the diagnosis of NASH ultimately requires a liver biopsy, which remains the gold standard for characterizing liver histology alterations. NAFL is described by convention, as the existence of 5% hepatic steatosis without hepatocyte ballooning, which is a sign of hepatocellular damage [40]. NAFL progresses to NASH when evidence of hepatocellular injury characterized by the presence of lobular inflammation (mixture of CD4-(+), CD8-(+) lymphocytes, Kupffer cell aggregates, polymorphonuclear leukocytes, macrophages, T-cells), hepatocellular ballooning degeneration, apoptotic bodies, and Mallory-Denk bodies (eosinophilic intracytoplasmic inclusion composed of misfolded filaments of keratins, heat-shock proteins) [41] can be seen on histology samples [17]. The diagnosis is challenging at times because the characteristics of steatohepatitis are not uniformly present in all biopsies. Early in the course of the illness, the histologic changes are distributed in a specific way, with the most severe changes occurring in acinar zone 3. Perisinusoidal/pericellular fibrosis, which features a “chicken wire” pattern is typical for NASH and is the most common type of fibrosis in NASH [42]. Therefore, it is crucial to apply generally accepted standards for the histological characterization. There are two accepted histological scoring system for the diagnosis. One is Steatosis Activity Fibrosis from the European Fatty Liver Inhibition of Progression Consortium which is comprised of three main characteristics (steatosis, activity, fibrosis) defined as SAF score based on which fatty liver inhibition of progression (FLIP) algorithm was developed, in order to decrease interobserver variations among pathologists [43]. The second score is developed by The Pathology Committee of the NASH Clinical Research Network and addresses the full spectrum of lesions of NAFDL and proposed a NAFLD activity score (NAS). Both scores are typically used to standardize the outcomes of clinical studies and employ the same histological lesions in the diagnosis of NASH; however, the SAF score also considers the phases of fibrosis as opposed to NAS, which does not include this feature because it is not reversible [44]. The NAS score can capture the histological response to therapy and utilize features of active injury that are at least potentially reversible. The score, which ranges from 0 to 8, is determined by adding the unweighted scores for ballooning (0–2), lobular inflammation (0–3), and steatosis (0–3). The use of a semiquantitative scale to stage fibrosis is a significant concern as well. Due to the scales not being linear, the clinical implications between consecutive phases are not equal (F0 to F1 is not equal to F2 to F3). More advanced stages of cirrhosis cannot be identified once the diagnosis of cirrhosis (F4) has been made [45].

## 7. Prognosis in MetS

MetS was reported to be associated with a greater risk of CVD mortality, especially in case of its independent components such as HBP and IFG [46]. MetS is frequently associated with T2DM, and, together with NAFLD, lead to a more rapid progression towards NASH [47]. Zhang et al. reported a 5-year survival rate for patients with NASH cirrhosis of 75% [48].

## 8. Non-Invasive Assessment of NAFLD

The ideal biomarker is a trait that can be objectively assessed, recapitulates typical pathogenic or physiological processes, and evolves in parallel with the improvements and worsening of the disease. Biomarkers can be utilized in NAFLD to diagnose and measure steatosis, assess for the existence of NASH, and quantify fibrosis. It is important to identify which patients are associating features that lead to the progression of liver disease because of the specific treatment and the high risk of developing cirrhosis and its complications, including liver-related mortality [49]. The most accurate method for diagnosing and staging NASH is liver biopsy, but this procedure is intrusive, expensive, fraught with potential risks, and characterized by interobserver variability; due to these disadvantages clinicians are developing several non-invasive modalities for diagnosing NASH and staging liver fibrosis. These techniques include predictive models, serum biomarkers, and imaging-based biomarkers [49].

### 8.1. Clinical and Laboratory Variables (Serum Biomarkers)

Assessment of steatosis: in clinical practice, scores for the identification of steatosis have not yet attracted significant attention because diagnosis of NAFLD using recommended imaging criteria is accurate [50]. Scores that can be used to determine steatosis include the Fatty Liver Index, Hepatic Steatosis Index, SteatoTest, NAFLD Liver Fat Score, and Index of NASH. These tests are comparable in terms of the accuracy of steatosis diagnosis. To find independent and quantitative markers of steatosis, more studies are required [51].

Assessment of fibrosis: AF in NAFLD is clinically predicted by male gender, Caucasian ethnicity, presence of T2DM, obesity, and elevated aspartate transaminase (AST) or alanine aminotransferase (ALT) levels [52]. AST is a better predictor than ALT and early studies found that an AST/ALT ratio >1 is associated with AF [53]. Serum ferritin level is associated with hepatic iron deposition and histologic activity and, therefore, it is considered an independent predictor of hepatic fibrosis among patients with NAFLD [54].

AST to platelet ratio index (APRI) takes into consideration the AST and platelets and combines two biological phenomena that happen when NAFL progress to NASH: the increase of AST and, respectively, the decrease in platelet count. It is a handy tool in clinical practice due to its simplicity. The sensitivities and specificities in predicting NAFLD with an APRI threshold of 1.0 and 1.5 were 50.0% and 84.0% and 18.3% and 96.1%, respectively, with a positive predictive value (PPV) of 34% and 56% and a negative predictive value (NPV) of 90% and 79% for AF as seen in Table 2 [55].

Fibrosis-4 (FIB-4) was initially created for patients with hepatitis C and HIV and, eventually, it was discovered that the test was helpful in NAFLD. It uses age, AST, ALT, and platelet count (using the same biological phenomena as APRI). At a value for FIB-4 1.93, the specificity is 90%, while the sensitivity is only 50% in identifying AF. A FIB-4 index of 1.93 had a 90% NPV and a 36% PPV, while a FIB4 of 2.67 had an 85% NPV and an 80% PPV for detecting AF, as mentioned in Table 2 [56].

NAFLD Fibrosis Score (NFS) is composed of 6 variables, and each of them represents an independent indicator of AF: age, hyperglycaemia, BMI, platelet count, albumin, and AST/ALT ratio. A score lower than −1.45 predicts the absence of AF F3-F4 (negative predictive value), whereas a score higher than 0.67 predicts AF (positive predictive value). For a value of 1.45, the sensitivity and specificity are 77% and 71% with PPV of 52% and NPV of 88% for detecting AF, as seen in Table 2. It is considered that by applying this score, 75% of liver biopsies could be avoided [57]. NFS is inaccurate in patients with asplenia and after a trans-jugular intrahepatic portosystemic shunt because it may improve thrombocytopenia associated with liver cirrhosis [58].

BARD Score is formed by the weighted sum of three variables (BMI, AST/ALT ratio and presence of DM). A BMI ≥ 28 = 1 point, AST/ALT ratio ≥ 0.8 = 2 points and T2DM = 1 point. A score of 2–4 was associated with 17-fold odds of F3–F4 stages of fibrosis and have a negative predictive value of 97%. For a cut-off of 2, the sensitivity and specificity for detecting AF are 86% and 72%, respectively, with a PPV of 35% and a NPV of 97%, as seen in Table 2 [59].

Fibro test (FT) employs age- and gender-corrected total bilirubin, gamma glutamyl transferase (GGT), 2-macroglobulin, apolipoprotein A1, and haptoglobin measurements. It is important to do a correct anamnesis when using this tool because other conditions interfering with alpha-2-macroglobulin (an inflammatory condition), bilirubin (Gilbert’s), haptoglobin (haemolysis), GGT (using alcohol), can alter the results and give an inaccurate estimation of fibrosis. A cut-off of 0.30 has a negative predictive value of 90% for AF (sensitivity 77%), while a cut-off of 0.70 have a 73% positive predictive value for AF (specificity 98%), as seen in Table 2 [60].

Enhanced liver fibrosis (ELF) is a more recently developed test, after demonstrating that eliminating age from the formula does not change the test’s usefulness. ELF is a more complex score and includes markers related to matrix turnover from serum samples: hyaluronic acid, amino-terminal propeptide of type III collagen, and tissue inhibitor of metalloproteinase 1. A score of 10.51 has a sensitivity of 100% and a specificity of 98% in detecting AF with a PPV of 80% and a NPV of 100%, as seen in Table 2. It can predict severe fibrosis and 81% of biopsies could have been avoided by using clinical utility modelling to predict severe fibrosis [61]. The ELF has also been validated in paediatric patients with NAFLD and it is important for identifying patients with progressive fibrosis that require further histopathological analysis and treatment monitoring [62]. The primary drawback of ELF is the tests’ comparatively poor accessibility in routine clinical practice [48].

### 8.2. Imaging-Based Biomarkers

Assessment of steatosis: The most extensively used, least expensive, well-tolerated, and secure method for grading and diagnosing steatosis (mild, moderate, and severe) is conventional US. Despite the great accuracy in the detection of parenchymal fat (when steatosis is less than 20 percent, it has low sensitivity and is unreliable [63]), several important limitations can be faced, including subjectivity, operator reliance, and some conditions like renal illness and morbid obesity [64]. In terms of tracking therapy progress over time, this approach is not very useful [51].

Controlled attenuation parameter (CAP) is a novel US-based technique using a process based on transient elastography (TE) for measuring steatosis. For a grade of steatosis of 5–33% the sensitivity is 73.1% and specificity is 95.2%, the cut-off 327 dB/m for detecting advance fibrosis has a sensitivity of 78% and a specificity of 84% with a PPV of 26% and a NPV of 98%, as seen in Table 2 [65]. This tool is limited among patients with severe obesity and with ascites [66].

Magnetic resonance imaging proton-density fat fraction (MRI-PDFF) is a trustworthy and validated marker of hepatic steatosis, measuring fat in the liver and monitoring patients for therapy efficacy evaluation [67]. The cost of the method, the level of assessment skill required, and other MRI-based restrictions are its disadvantages [68].

Assessment of fibrosis: The techniques, which mostly rely on US and magnetic resonance technologies, are more precise than blood biomarkers but more difficult to use and more expensive in clinical settings [66].

**Figure 1:**
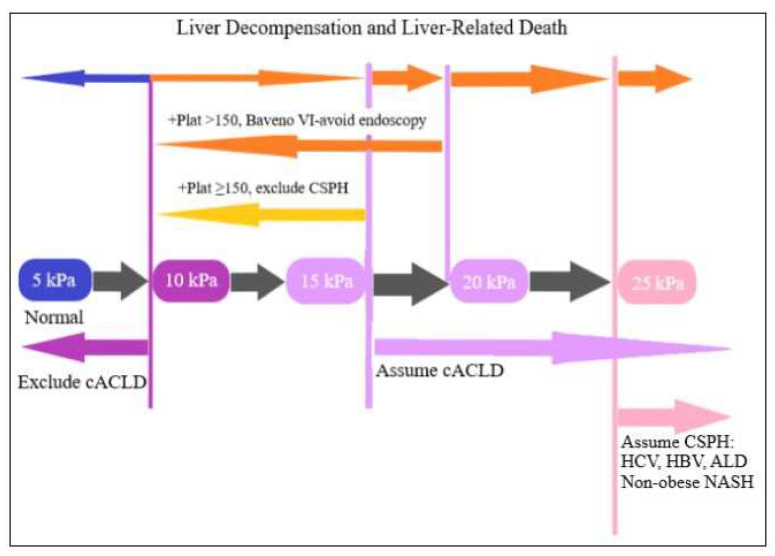
Representation of Baveno VII criteria.

TE; Fibroscan or vibration controlled TE is a US-based method for assessing liver elasticity by using the TE technique [67]. The main clinical indication is fibrosis staging and therefore the liver elasticity is related to liver fibrosis. A mechanical vibrating source is applied in the area dullest to percussion (9th–11th intercostal space) and an area of the liver that is about 6 cm deep is examined. At least ten reliable measures should be taken by the US operator. This is a reliable technique for identifying F4 fibrosis (cirrhosis) and is useful for differentiating between AF (F2 or grater) and minimal or no fibrosis [69]. Patients are recommended to fast for three hours prior to the measurement because results may be influenced by fed state [70]. The test results are reported in kilopascal (kPa) units [69]. The latest cut-off values reported are from Baveno VII, respectively, 5–10 kPa for exclusion of compensated advanced chronic liver disease (cACLD), 15–25 kPa for assumed cACLD and over 25 kPa for assumed clinically significant portal hypertension as seen in Figure 1 [70]. For a cut-off value of 25 kPa, the specificity is 90.4% with a PPV of 91.5% as seen in Table 2 [71,72].

Acoustic radiation force impulse imaging (ARFI) is also a recently developed, US-based technique, which applies vibration to evaluate the stiffness of the tissue by wave speed propagation to estimate liver fibrosis. ARFI is integrated in the regular US device. The short-duration acoustic push pulses induce within tissues shear stress, with intensities depending upon tissue attenuation. The shear stress generates shear waves that propagate, perpendicular to the main US beam, away from the original region of excitation [73]. As the US detector in 1D, ARFI does not offer morphologic imaging guidance. ARFI diagnostic accuracy is comparable to TE in assessing liver fibrosis [74]. The cut-off values for diagnosis of fibrosis varies among studies and vendor system. For a value of 1.29 (m/s), the sensitivity is 91%, the specificity is 92%, for detecting advance fibrosis with a PPV of 93% and an NPV of 90%, as seen in Table 2 [75].

Shear wave elastography (SWE), also known as two-dimensional SWE, is a US-based technique that measure liver stiffness, such as TE, in kPa units, with the advantage of assessment of hepatic parenchyma. At a cut-off of 3 kPa, it shows a specificity of 92% and a sensitivity of 90% in diagnosis of AF with a PPV of 88% and a NPV of 93%, as seen in Table 2 [55,76,77].

Magnetic resonance elastography (MRE) is an advanced MR-based technique which can also be performed to measure liver fibrosis. MRE is performed on a regular MRI scanner which has additional hardware and software installed. Mechanical waves at 60 Hz are generated in the liver by a passive driver attached to the body, anterior to the liver [78]. The passive driver is coupled to an active acoustic driver located outside the scanner room. At the end of the examination, the machine provides a color-coded stiffness map which interrogates the entire liver [78]. The accuracy of MRE in the diagnosis of AF is independent of BMI, and degree of inflammation (ascites), and therefore has an advantage over TE [79]. In detecting AF for a cut-off of 3.6 kPa, MRE has a sensitivity of 86%, a specificity of 91% with a PPV of 68% and an NPV of 97%, as seen in Table 2. MRE has a sensitivity of 91% and a specificity of 81% for an optimal cut-off of 4.11 kPa in detecting cirrhosis [80]. Regarding prospective patient follow-up, MRE can evaluate regression, treatment response, and progression during the disease or in response to therapy [81].

## 9. Conclusions

MetS fundamentally represents an association of CVD and T2DM risk factors, characterized by IR. NAFLD can be a cause, but also a consequence of IR and, therefore, it is frequently associated with Mets. NAFL, in association with MetS and T2DM, increases the progression to NASH, augmenting the morbidity and mortality rates. An early diagnosis of NAFLD is a key for an optimal treatment of these patients, in order to minimize their morbidity. Liver biopsy is the gold standard for diagnosis of NAFLD, but because it is invasive, novel developed non-invasive techniques, which use serum biomarkers, and imaging-based biomarkers should be promoted and used on a larger scale, respectively, APRI, FIB-4, NFS, BARD SCORE, FT, ELF, TE, ARFI, SWE and MRE.

## Figures and Tables

**Table 2:** Non-invasive assessment of advanced fibrosis in NAFLD. Cut-off values, sensitivity, specificity, PPV, NPV.

Non-Invasive Assessment of Advanced Fibrosis in NAFLD	Cut-Off Values for Advanced Fibrosis	Sensitivity (%)	Specificity (%)	PPV (%)	NPV (%)
Serum Biomarkers					
APRI	1.0 1.5	50 18	84 90	34 56	90 79
FIB-4	1.93 2.67	50 34	90 98	36 77	94 85
NFS	−1.45 0.67	77 43	71 96	52 82	88 80
BARD Score	2	86	72	35	97
FT	0.3 0.7	77 15	77 98	54 73	90 76
ELF	10.51	100	98	80	100
Imaging-Based Biomarkers					
CAP	327 (dB/m)	78	84	26	98
TE	25 (kPa)	99.5	90.4	91.5	98.6
ARFI	1.29 (m/s)	91	92	93	90
SWE	3 (kPa)	90	92	88	93
MRE	3.6 (kPa)	86	91	68	97

APRI, AST to platelet ratio index; FIB-4, fibrosis-4; NFS, NAFLD fibrosis score; FT, fibro test; ELF, enhanced liver fibrosis; CAP, controlled attenuation parameter; TE, transient elastography; ARFI, acoustic radiation force impulse imaging; SWE, shear wave elastography; MRE, magnetic resonance elastography; PPV, positive predictive value; NPV, negative predictive value.

## Data Availability

Not applicable.

## References

[1] Barale C., Russo I. (2020). Influence of Cardiometabolic Risk Factors on Platelet Function. Int. J. Mol. Sci..

[2] Lemieux I., Després J.-P. (2020). Metabolic Syndrome: Past, Present and Future. Nutrients.

[3] Grander C., Grabherr F., Moschen A.R., Tilg H. (2016). Non-Alcoholic Fatty Liver Disease: Cause or Effect of Metabolic Syndrome. Visc. Med..

[4] Riazi K., Azhari H., Charette J.H., Underwood F.E., King J.A., Afshar E.E., Swain M.G., Congly S.E., Kaplan G.G., Shaheen A.-A. (2022). The prevalence and incidence of NAFLD worldwide: A systematic review and meta-analysis. Lancet Gastroenterol. Hepatol..

[5] Jackson S.E., Llewellyn C.H., Smith L. (2020). The obesity epidemic—Nature via nurture: A narrative review of high-income countries. SAGE Open Med..

[6] Choudhary N.S., Duseja A. (2019). Screening of Cardiovascular Disease in Nonalcoholic Fatty Liver Disease: Whom and How?. J. Clin. Exp. Hepatol..

[7] Berg E.H.V.D., Douwes R.M., de Meijer V.E., Schreuder T.C., Blokzijl H. (2018). Liver transplantation for NASH cirrhosis is not performed at the expense of major post-operative morbidity. Dig. Liver Dis..

[8] Cleeman J., Grundy S., Becker D., Clark L. (2001). Expert panel on detection, evaluation and treatment of high blood cholesterol in adults. Executive summary of the third report of the National Cholesterol Education Program (NCEP) Adult Treatment Panel (ATP III). JAMA.

[9] Kassi E., Pervanidou P., Kaltsas G., Chrousos G. (2011). Metabolic syndrome: Definitions and controversies. BMC Med..

[10] Grundy S.M., Cleeman J.I., Daniels S.R., Donato K.A., Eckel R.H., Franklin B.A. (2005). Diagnosis and management of the metabolic syndrome: An American Heart Association/National Heart, Lung, and Blood Institute scientific statement. Circulation.

[11] Noubiap J.J., Nansseu J.R., Lontchi-Yimagou E., Nkeck J.R., Nyaga U.F., Ngouo A.T., Tounouga D.N., Tianyi F.L., Foka A.J., Ndoadoumgue A.L. (2022). Geographic distribution of metabolic syndrome and its components in the general adult population: A meta-analysis of global data from 28 million individuals. Diabetes Res. Clin. Pract..

[12] Patel P., Abate N. (2013). Role of Subcutaneous Adipose Tissue in the Pathogenesis of Insulin Resistance. J. Obes..

[13] Ramírez-Manent J.I., Jover A.M., Martinez C.S., Tomás-Gil P., Martí-Lliteras P., López-González A. (2023). Waist Circumference Is an Essential Factor in Predicting Insulin Resistance and Early Detection of Metabolic Syndrome in Adults. Nutrients.

[14] Szczepaniak L.S., Nurenberg P., Leonard D., Browning J.D., Reingold J.S., Grundy S., Hobbs H.H., Dobbins R.L. (2005). Magnetic resonance spectroscopy to measure hepatic triglyceride content: Prevalence of hepatic steatosis in the general population. Am. J. Physiol. Metab..

[15] Yki-Järvinen H. (2005). Fat in the liver and insulin resistance. Ann. Med..

[16] Busnatu S.-S., Salmen T., Pana M.-A., Rizzo M., Stallone T., Papanas N., Popovic D., Tanasescu D., Serban D., Stoian A.P. (2022). The Role of Fructose as a Cardiovascular Risk Factor: An Update. Metabolites.

[17] Chalasani N., Younossi Z., LaVine J.E., Diehl A.M., Brunt E.M., Cusi K., Charlton M., Sanyal A.J. (2012). The diagnosis and management of non-alcoholic fatty liver disease: Practice Guideline by the American Association for the Study of Liver Diseases, American College of Gastroenterology, and the American Gastroenterological Association. Hepatology.

[18] Yki-Järvinen H. (2014). Non-alcoholic fatty liver disease as a cause and a consequence of metabolic syndrome. Lancet Diabetes Endocrinol..

[19] Browning J.D., Szczepaniak L.S., Dobbins R., Nuremberg P., Horton J.D., Cohen J.C., Grundy S.M., Hobbs H.H. (2004). Prevalence of hepatic steatosis in an urban population in the United States: Impact of ethnicity. Hepatology.

[20] Hashimoto E., Tokushige K., Ludwig J. (2014). Diagnosis and classification of non-alcoholic fatty liver disease and non-alcoholic steatohepatitis: Current concepts and remaining challenges. Hepatol. Res..

[21] Bellentani S. (2017). The epidemiology of non-alcoholic fatty liver disease. Liver Int..

[22] Charlton M.R., Burns J.M., Pedersen R.A., Watt K.D., Heimbach J.K., Dierkhising R.A. (2011). Frequency and Outcomes of Liver Transplantation for Nonalcoholic Steatohepatitis in the United States. Gastroenterology.

[23] Anstee Q.M., Targher G., Day C.P. (2013). Progression of NAFLD to diabetes mellitus, cardiovascular disease or cirrhosis. Nat. Rev. Gastroenterol. Hepatol..

[24] Younossi Z., Stepanova M., Ong J.P., Jacobson I.M., Bugianesi E., Duseja A., Eguchi Y., Wong V.W., Negro F., Yilmaz Y. (2018). Nonalcoholic Steatohepatitis Is the Fastest Growing Cause of Hepatocellular Carcinoma in Liver Transplant Candidates. Clin. Gastroenterol. Hepatol..

[25] Thong V.D., Quynh B.T.H. (2021). Correlation of Serum Transaminase Levels with Liver Fibrosis Assessed by Transient Elastography in Vietnamese Patients with Nonalcoholic Fatty Liver Disease. Int. J. Gen. Med..

[26] Fedeli U., Avossa F., Ferroni E., De Paoli A., Donato F., Corti M.C. (2019). Prevalence of chronic liver disease among young/middle-aged adults in Northern Italy: Role of hepatitis B and hepatitis C virus infection by age, sex, ethnicity. Heliyon.

[27] Kitazawa A., Maeda S., Fukuda Y. (2021). Fatty liver index as a predictive marker for the development of diabetes: A retrospective cohort study using Japanese health check-up data. PLoS ONE.

[28] Cotter T.G., Rinella M. (2020). Nonalcoholic Fatty Liver Disease 2020: The State of the Disease. Gastroenterology.

[29] Younossi Z.M., Henry L. (2021). Epidemiology of non-alcoholic fatty liver disease and hepatocellular carcinoma. JHEP Rep..

[30] Stepanova M., Rafiq N., Makhlouf H., Agrawal R., Kaur I., Younoszai Z., McCullough A., Goodman Z., Younossi Z.M. (2013). Predictors of All-Cause Mortality and Liver-Related Mortality in Patients with Non-Alcoholic Fatty Liver Disease (NAFLD). Dig. Dis. Sci..

[31] Montemayor S., Bouzas C., Mascaró C.M., Casares M., Llompart I., Abete I., Angullo-Martinez E., Zulet M., Martínez J.A., Tur J.A. (2022). Effect of Dietary and Lifestyle Interventions on the Amelioration of NAFLD in Patients with Metabolic Syndrome: The FLIPAN Study. Nutrients.

[32] Konieczna J., Fiol M., Colom A., Martínez-González M., Salas-Salvadó J., Corella D., Soria-Florido M.T., Martínez J.A., Alonso-Gómez M., Wärnberg J. (2022). Does Consumption of Ultra-Processed Foods Matter for Liver Health? Prospective Analysis among Older Adults with Metabolic Syndrome. Nutrients.

[33] van Kleef L.A., Hofman A., Voortman T., de Knegt R.J. (2021). Objectively Measured Physical Activity Is Inversely Associated with Nonalcoholic Fatty Liver Disease: The Rotterdam Study. Am. J. Gastroenterol..

[34] Lassailly G., Caiazzo R., Ntandja-Wandji L.-C., Gnemmi V., Baud G., Verkindt H., Ningarhari M., Louvet A., Leteurtre E., Raverdy V. (2020). Bariatric Surgery Provides Long-term Resolution of Nonalcoholic Steatohepatitis and Regression of Fibrosis. Gastroenterology.

[35] Pedersen J.S., Rygg M.O., Serizawa R.R., Kristiansen V.B., Albrechtsen N.J.W., Gluud L.L., Madsbad S., Bendtsen F. (2021). Effects of Roux-en-Y Gastric Bypass and Sleeve Gastrectomy on Non-Alcoholic Fatty Liver Disease: A 12-Month Follow-Up Study with Paired Liver Biopsies. J. Clin. Med..

[36] Newsome P.N., Buchholtz K., Cusi K., Linder M., Okanoue T., Ratziu V., Sanyal A.J., Sejling A.-S., Harrison S.A. (2021). A Placebo-Controlled Trial of Subcutaneous Semaglutide in Nonalcoholic Steatohepatitis. N. Engl. J. Med..

[37] Salmen T., Pietroșel V.-A., Mihai B.-M., Bica I.C., Teodorescu C., Păunescu H., Coman O.A., Mihai D.-A., Stoian A.P. (2022). Non-Insulin Novel Antidiabetic Drugs Mechanisms in the Pathogenesis of COVID-19. Biomedicines.

[38] Mirarchi L., Amodeo S., Citarrella R., Licata A., Soresi M., Giannitrapani L. (2022). SGLT2 Inhibitors as the Most Promising Influencers on the Outcome of Non-Alcoholic Fatty Liver Disease. Int. J. Mol. Sci..

[39] Vilar-Gomez E., Vuppalanchi R., Gawrieh S., Ghabril M., Saxena R., Cummings O.W., Chalasani N. (2018). Vitamin E Improves Transplant-Free Survival and Hepatic Decompensation among Patients with Nonalcoholic Steatohepatitis and Advanced Fibrosis. Hepatology.

[40] Kleiner D.E., Brunt E.M., Van Natta M., Behling C., Contos M.J., Cummings O.W., Ferrell L.D., Liu Y.-C., Torbenson M.S., Unalp-Arida A. (2005). Design and validation of a histological scoring system for nonalcoholic fatty liver disease. Hepatology.

[41] Zatloukal K., French S.W., Stumptner C., Strnad P., Harada M., Toivola D.M. (2007). From Mallory to Mallory–Denk bodies: What, how and why?. Exp. Cell Res..

[42] Brown G.T., Kleiner D.E. (2016). Histopathology of nonalcoholic fatty liver disease and nonalcoholic steatohepatitis. Metabolism.

[43] Bedossa P., FLIP Pathology Consortium (2014). Utility and appropriateness of the fatty liver inhibition of progression (FLIP) algorithm and steatosis, activity, and fibrosis (SAF) score in the evaluation of biopsies of nonalcoholic fatty liver disease. Hepatology.

[44] Kleiner D.E., Bedossa P. (2015). Liver Histology and Clinical Trials for Nonalcoholic Steatohepatitis-Perspectives From 2 Pathologists. Gastroenterology.

[45] Duarte-Rojo A., Altamirano J.T., Feld J.J. (2012). Noninvasive markers of fibrosis: Key concepts for improving accuracy in daily clinical practice. Ann. Hepatol..

[46] Mazloomzadeh S., Karami Zarandi F., Shoghli A., Dinmohammadi H. (2019). Metabolic Syndrome, Its Components and Mortality: A Population-Based Study. Med. J. Islam. Repub. Iran.

[47] Cariou B., Byrne C.D., Loomba R., Sanyal A.J. (2021). Nonalcoholic fatty liver disease as a metabolic disease in humans: A literature review. Diabetes Obes. Metab..

[48] Li B., Zhang C., Zhan Y.-T. (2018). Nonalcoholic Fatty Liver Disease Cirrhosis: A Review of Its Epidemiology, Risk Factors, Clinical Presentation, Diagnosis, Management, and Prognosis. Can. J. Gastroenterol. Hepatol..

[49] Younossi Z.M., Loomba R., Anstee Q.M., Rinella M.E., Bugianesi E., Marchesini G., Neuschwander-Tetri B.A., Serfaty L., Negro F., Caldwell S.H. (2017). Diagnostic modalities for nonalcoholic fatty liver disease, nonalcoholic steatohepatitis, and associated fibrosis. Hepatology.

[50] Stern C., Castera L. (2016). Non-invasive diagnosis of hepatic steatosis. Hepatol. Int..

[51] Fedchuk L., Nascimbeni F., Pais R., Charlotte F., Housset C., Ratziu V., the LIDO Study Group (2014). Performance and limitations of steatosis biomarkers in patients with nonalcoholic fatty liver disease. Aliment. Pharmacol. Ther..

[52] Hossain N., Afendy A., Stepanova M., Nader F., Srishord M., Rafiq N., Goodman Z., Younossi Z. (2009). Independent Predictors of Fibrosis in Patients with Nonalcoholic Fatty Liver Disease. Clin. Gastroenterol. Hepatol..

[53] Verma S., Jensen D., Hart J., Mohanty S.R. (2013). Predictive value of ALT levels for non-alcoholic steatohepatitis (NASH) and advanced fibrosis in non-alcoholic fatty liver disease (NAFLD). Liver Int..

[54] Kowdley K.V., Belt P., Wilson L.A., Yeh M.M., Neuschwander-Tetri B.A., Chalasani N., Sanyal A.J., Nelson J.E., the NASH Clinical Research Network (2012). Serum ferritin is an independent predictor of histologic severity and advanced fibrosis in patients with nonalcoholic fatty liver disease. Hepatology.

[55] Xiao G., Zhu S., Xiao X., Yan L., Yang J., Wu G. (2017). Comparison of laboratory tests, ultrasound, or magnetic resonance elastography to detect fibrosis in patients with nonalcoholic fatty liver disease: A meta-analysis. Hepatology.

[56] Shah A.G., Lydecker A., Murray K., Tetri B.N., Contos M.J., Sanyal A.J., Nash Clinical Research Network (2009). Comparison of Noninvasive Markers of Fibrosis in Patients with Nonalcoholic Fatty Liver Disease. Clin. Gastroenterol. Hepatol..

[57] Angulo P., Hui J.M., Marchesini G., Bugianesi E., George J., Farrell G.C., Enders F., Saksena S., Burt A.D., Bida J.P. (2007). The NAFLD fibrosis score: A noninvasive system that identifies liver fibrosis in patients with NAFLD. Hepatology.

[58] Massoud O.I., Zein N.N. (2017). The Effect of Transjugular Intrahepatic Portosystemic Shunt on Platelet Counts in Patients with Liver Cirrhosis. Gastroenterol. Hepatol..

[59] Raszeja-Wyszomirska J., Szymanik B., Ławniczak M., Kajor M., Chwist A., Milkiewicz P., Hartleb M. (2010). Validation of the BARD scoring system in Polish patients with nonalcoholic fatty liver disease (NAFLD). BMC Gastroenterol..

[60] Ratziu V., Massard J., Charlotte F., Messous D., Imbert-Bismut F., Bonyhay L. (2006). Diagnostic value of biochemical markers (FibroTest-FibroSURE) for the prediction of liver fibrosis in patients with non-alcoholic fatty liver disease. BMC Gastroenterol..

[61] Parkes J., Guha I.N., Roderick P., Harris S., Cross R., Manos M.M., Irving W., Zaitoun A., Wheatley M., Ryder S. (2010). Enhanced Liver Fibrosis (ELF) test accurately identifies liver fibrosis in patients with chronic hepatitis C. J. Viral Hepat..

[62] Nobili V., Parkes J., Bottazzo G., Marcellini M., Cross R., Newman D., Vizzutti F., Pinzani M., Rosenberg W.M. (2009). Performance of ELF Serum Markers in Predicting Fibrosis Stage in Pediatric Non-Alcoholic Fatty Liver Disease. Gastroenterology.

[63] Younossi Z.M., Golabi P., de Avila L., Paik J.M., Srishord M., Fukui N., Qiu Y., Burns L., Afendy A., Nader F. (2019). The global epidemiology of NAFLD and NASH in patients with type 2 diabetes: A systematic review and meta-analysis. J. Hepatol..

[64] Almeida A.D.M., Cotrim H.P., Barbosa D.B.V., De Athayde L.G.M., Santos A.S., Bitencourt A.G.V., De Freitas L.A.R., Rios A., Alves E. (2008). Fatty liver disease in severe obese patients: Diagnostic value of abdominal ultrasound. World J. Gastroenterol..

[65] Chon Y.E., Jung K.S., Kim S.U., Park J.Y., Park Y.N., Kim D.Y., Ahn S.H., Chon C.Y., Lee H.W., Park Y. (2013). Controlled attenuation parameter (CAP) for detection of hepatic steatosis in patients with chronic liver diseases: A prospective study of a native Korean population. Liver Int..

[66] Altamirano J., Qi Q., Choudhry S., Abdallah M., Singal A.K., Humar A., Bataller R., Borhani A.A., Duarte-Rojo A. (2020). Non-invasive diagnosis: Non-alcoholic fatty liver disease and alcoholic liver disease. Transl. Gastroenterol. Hepatol..

[67] Diaconu C.-T., Guja C. (2022). Nonalcoholic Fatty Liver Disease and Its Complex Relation with Type 2 Diabetes Mellitus—From Prevalence to Diagnostic Approach and Treatment Strategies. J. Clin. Med..

[68] Ajmera V., Park C.C., Caussy C., Singh S., Hernandez C., Bettencourt R., Hooker J., Sy E., Behling C., Xu R. (2018). Magnetic Resonance Imaging Proton Density Fat Fraction Associates with Progression of Fibrosis in Patients with Nonalcoholic Fatty Liver Disease. Gastroenterology.

[69] Castéra L., Vergniol J., Foucher J., Le Bail B., Chanteloup E., Haaser M., Darriet M., Couzigou P., de Lédinghen V. (2005). Prospective comparison of transient elastography, Fibrotest, APRI, and liver biopsy for the assessment of fibrosis in chronic hepatitis C. Gastroenterology.

[70] Vuppalanchi R., Weber R., Russell S., Gawrieh S., Samala N., Slaven J.E., Harden L., Chalasani N. (2019). Is Fasting Necessary for Individuals with Nonalcoholic Fatty Liver Disease to Undergo Vibration-Controlled Transient Elastography?. Am. J. Gastroenterol..

[71] Jachs M., Hartl L., Simbrunner B., Bauer D., Paternostro R., Scheiner B., Balcar L., Semmler G., Stättermayer A.F., Pinter M. (2022). The Sequential Application of Baveno VII Criteria and VITRO Score Improves Diagnosis of Clinically Significant Portal Hypertension. Clin. Gastroenterol. Hepatol..

[72] de Franchis R., Bosch J., Garcia-Tsao G., Reiberger T., Ripoll C., Baveno VII Faculty (2022). Baveno VII—Renewing consensus in portal hypertension. J. Hepatol..

[73] Bruno C., Minniti S., Bucci A., Mucelli R.P. (2016). ARFI: From basic principles to clinical applications in diffuse chronic disease—A review. Insights Imaging.

[74] Friedrich-Rust M., Wunder K., Kriener S., Sotoudeh F., Richter S., Bojunga J., Herrmann E., Poynard T., Dietrich C.F., Vermehren J. (2009). Liver Fibrosis in Viral Hepatitis: Noninvasive Assessment with Acoustic Radiation Force Impulse Imaging versus Transient Elastography. Radiology.

[75] Cassinotto C., Boursier J., de Lédinghen V., Lebigot J., Lapuyade B., Cales P., Hiriart J.B., Michalak S., Bail B.L., Cartier V. (2016). Liver stiffness in nonalcoholic fatty liver disease: A comparison of supersonic shear imaging, FibroScan, and ARFI with liver biopsy. Hepatology.

[76] Jiang W., Huang S., Teng H., Wang P., Wu M., Zhou X., Ran H. (2018). Diagnostic accuracy of point shear wave elastography and transient elastography for staging hepatic fibrosis in patients with non-alcoholic fatty liver disease: A meta-analysis. BMJ Open.

[77] Cui J., Heba E., Hernandez C., Haufe W., Hooker J., Andre M.P. (2016). MRE is superior to ARFI for the diagnosis of fibrosis in patients with biopsy-proven NAFLD: A prospective study. Hepatology.

[78] Kim D., Kim W.R., Talwalkar J.A., Kim H.J., Ehman R.L. (2013). Advanced Fibrosis in Nonalcoholic Fatty Liver Disease: Noninvasive Assessment with MR Elastography. Radiology.

[79] Singh S., Venkatesh S.K., Loomba R., Wang Z., Sirlin C., Chen J., Yin M., Miller F.H., Low R.N., Hassanein T. (2015). Magnetic resonance elastography for staging liver fibrosis in non-alcoholic fatty liver disease: A diagnostic accuracy systematic review and individual participant data pooled analysis. Eur. Radiol..

[80] Singh S., Venkatesh S.K., Wang Z., Miller F.H., Motosugi U., Low R.N., Hassanein T., Asbach P., Godfrey E.M., Yin M. (2014). Diagnostic Performance of Magnetic Resonance Elastography in Staging Liver Fibrosis: A Systematic Review and Meta-analysis of Individual Participant Data. Clin. Gastroenterol. Hepatol..

[81] Loomba R., Wolfson T., Ang B., Hooker J., Behling C., Peterson M., Valasek M., Lin G., Brenner D., Gamst A. (2014). Magnetic Resonance Elastography Predicts Advanced Fibrosis in Patients with Nonalcoholic Fatty Liver Disease: A Prospective Study. Hepatology.

